# Supplementary material for: Steady-State PERG Adaptation Reveals Temporal Abnormalities of Retinal Ganglion Cells in Treated Ocular Hypertension and Glaucoma
Source: Diagnostics (Basel). 2025 Jul 16;15(14):1797. doi: 10.3390/diagnostics15141797 (PMC12293091; doi:10.3390/diagnostics15141797)
Supplement: Supplementary file 1 [file diagnostics-15-01797-s001.zip › diagnostics-3726397-supplementary.pdf]

Supplementary Materials

Table S1. The table shows Pearson correlation coefficients between PERG parameters and structural/functional clinical variables.

Normal subjects

	IOP	MD	PSD	GCIPL	RNFL	A	DA	DELTA
**IOP**	1.00	0.25	-0.07	0.10	0.06	-0.12	0.02	0.15
**MD30.2**	0.25	1.00	-0.57**	-0.06	-0.17	0.06	0.33	0.16
**PSD30.2**	-0.07	-0.57**	1.00	0.75	-0.04	0.40	-0.18	-0.41
**GCIPL**	0.10	-0.06	0.75	1.00	0.80	0.33*	-0.10	-0.18
**RNFL**	0.06	-0.17	-0.04	0.80	1.00	0.27	-0.19	0.12
**A**	-0.12	0.06	0.40	0.33*	0.27	1.00	-0.63**	-0.16
**DA**	0.02	0.33	-0.18	-0.10	-0.19	-0.63**	1.00	0.21
**DELTA**	0.15	0.16	-0.41	-0.18	0.12	-0.16	0.21	1.00

OHT

	IOP	MD	PSD	GCIPL	RNFL	A	DA	DELTA
**IOP**	1.00	-0.32	0.14	-0.18	-0.20	0.41	-0.63**	0.08
**MD30.2**	-0.32	1.00	-0.16	0.02	0.05	0.07	0.16	-0.00
**PSD30.2**	0.14	-0.16	1.00	0.15	0.04	0.01	-0.14	0.05
**GCIPL**	-0.18	0.02	0.15	1.00	0.41	0.10	0.19	-0.21
**RNFL**	-0.20	0.05	0.04	0.41	1.00	0.14	-0.10	-0.05
**A**	0.41	0.07	0.01	0.10	0.14	1.00	-0.61**	-0.79**
**DA**	-0.63**	0.16	-0.14	0.19	-0.10	-0.61**	1.00	0.02
**DELTA**	0.08	-0.00	0.05	-0.21	-0.05	-0.79**	0.02	1.00

OAG

	IOP	MD	PSD	GCIPL	RNFL	A	DA	DELTA
**IOP**	1.00	0.11	0.09	0.34	0.16	0.21	-0.37	-0.27
**MD30.2**	0.11	1.00	-0.65**	0.33	0.17	-0.36	-0.05	0.31
**PSD30.2**	0.09	-0.65**	1.00	-0.16	0.12	0.41	-0.09	-0.39
**GCIPL**	0.34	0.33	-0.16	1.00	0.22	0.09	-0.31	0.15
**RNFL**	0.16	0.17	0.12	0.22	1.00	0.55	-0.10	0.28
**A**	0.21	-0.36	0.41	0.09	0.55	1.00	-0.68**	-0.72**
**DA**	-0.37	-0.05	-0.09	-0.31	-0.10	-0.68**	1.00	0.61**
**DELTA**	-0.27	0.31	-0.39	0.15	0.28	-0.72**	0.61**	1.00

* Correlation is significant at the 0.05 level (2-tailed); ** Correlation is significant at the 0.01 level (2-tailed).
